# Skull shape abnormalities in ischemic cerebrovascular and mental diseases in adults

**DOI:** 10.1038/s41598-021-97054-4

**Published:** 2021-09-02

**Authors:** Masaya Nagaishi, Yoshiko Fujii, Yoshiki Sugiura, Kensuke Suzuki

**Affiliations:** Department of Neurosurgery, Dokkyo Medical University Saitama Medical Center, 2-1-50 Minami-Koshigaya, Koshigaya-shi, Saitama 343-8555 Japan

**Keywords:** Neurological disorders, Neurology, Pathogenesis, Neurological disorders

## Abstract

Morphological changes in the child skull due to mechanical and metabolic stimulation and synostosis of the suture are well known. On the other hand, few studies have focused on clinical conditions relevant for adult skull deformity. We retrospectively reviewed computed tomography (CT) findings obtained from 365 cases that were treated for head injuries, moyamoya disease, cervical internal carotid artery stenosis, and mental diseases, and investigated the morphological changes in the skull associated with these diseases. The findings from head injuries were used not only for control subjects, but also for the analysis of generational changes in skull shape based on birth year. Head shape had a brachiocephalic tendency with occipital flattening in people born from the 1950s onwards. Cases of moyamoya disease, cervical internal carotid artery stenosis, and mental diseases showed significantly thicker frontal and occipital bone than those of control subjects. The skull thickening was especially noticeable in the frontal bone in moyamoya disease. Plagiocephaly was significantly frequent in moyamoya disease. These uncommon skull shapes are useful CT findings in screening subjects for early evidence of mental diseases and intracranial ischemic diseases with arterial stenosis.

## Introduction

The growth rate of the head’s circumference is related to cranial volume. Aberrant growth of cranial volume occurs in first 2 years of age, and the cranial volume at 9 years of age reaches 95% of that of the adult brain^1^. Head circumference continues to increase several years after the brain volume reaches a plateau, due to changes in skull thickness, reaching an adult morphological plateau at around 20 years age. Although mechanical and metabolic stimulation in childhood can effect structural changes in the skull, it commonly does not change in size and form after the head circumstance reaches a plateau.

The skull is formed both by intramembranous and endochondral ossification. The neurocranium mainly develops via intramembranous ossification, but ethmoid bone and portions of the occipital, temporal, and sphenoid bones develop via endochondral ossification. Bone remodeling is an important process for bone development and calcium homeostasis, and persists even after skeletal maturity^2^. Skeletal vasculature, mainly consisting of external carotid artery branches, plays an important role in these ossifications and bone remodeling^3^. A chronic reduction of cerebral blood flow via intracranial artery stenosis often leads to anastomotic connections between the external carotid artery and intracranial artery branches, which results in an increase in blood circulation in the skull^4^. Moyamoya disease is a chronic cerebrovascular occlusive disease showing slow progression that typically affects children under 20 years of age, as well as adults^5^. On the other hand, cervical internal carotid artery (ICA) stenosis is also a chronic atherosclerotic disease related to several risk factors, including hypertension, hyperlipidemia, and diabetes, and which commonly affects adults. Moyamoya disease and cervical ICA stenosis are responsible for a chronic reduction in cerebral blood flow and the subsequent gain of external blood flow in children and adults, respectively^6,7^. Additionally, chronic reduction in cerebral blood flow is observed in several mental diseases and associated with various psychotic symptoms^8^.

Plagiocephaly is characterized by an asymmetry in head shape due to positioning or synostosis of a cranial suture. Positional plagiocephaly is commonly developed when babies keep their head in same position and is frequently seen in the occipital part due to maintaining a continuous supine position (also known as flat head syndrome). Several studies have revealed cognitive, mental, or psychomotor developmental delays in patients with deformational plagiocephaly^9–13^. Although deformational plagiocephaly is one of the markers of developmental delay, it is not conclusive if these disabilities will persist in later life. Most studies have investigated head shape deformity and related factors in early childhood, and few studies have focused on the adult skull.

The aim of this study was to determine whether chronic reduction of cerebral blood flow in adulthood or childhood influences cranial shape deformities or cranial shape variation in adults with moyamoya disease and ICA stenosis. In addition, the presence of plagiocephaly was assessed in adults with mental diseases. We also investigated changes in skull shape by age group in the Japanese population.

## Materials and methods

We retrospectively reviewed clinical and CT findings obtained from 365 cases that were treated at the Dokkyo Medical University Saitama Medical Center from 2008 to 2020, analyzing adult skull shape and size with regard to birth year and primary diseases. Cases aged 20 years and older at the time of the CT scan were included. One hundred and sixty cases were included in this study and consisted of 20 consecutive cases with head injuries in the 1920–1990s, 50 cases with moyamoya disease, 50 cases with cervical ICA stenosis requiring surgery, and 105 cases with mental diseases (50 depression, 34 schizophrenia, and 21 anxiety cases). All patients were Japanese and those with neurocognitive disorders were excluded from this analysis.

**Figure 1:**
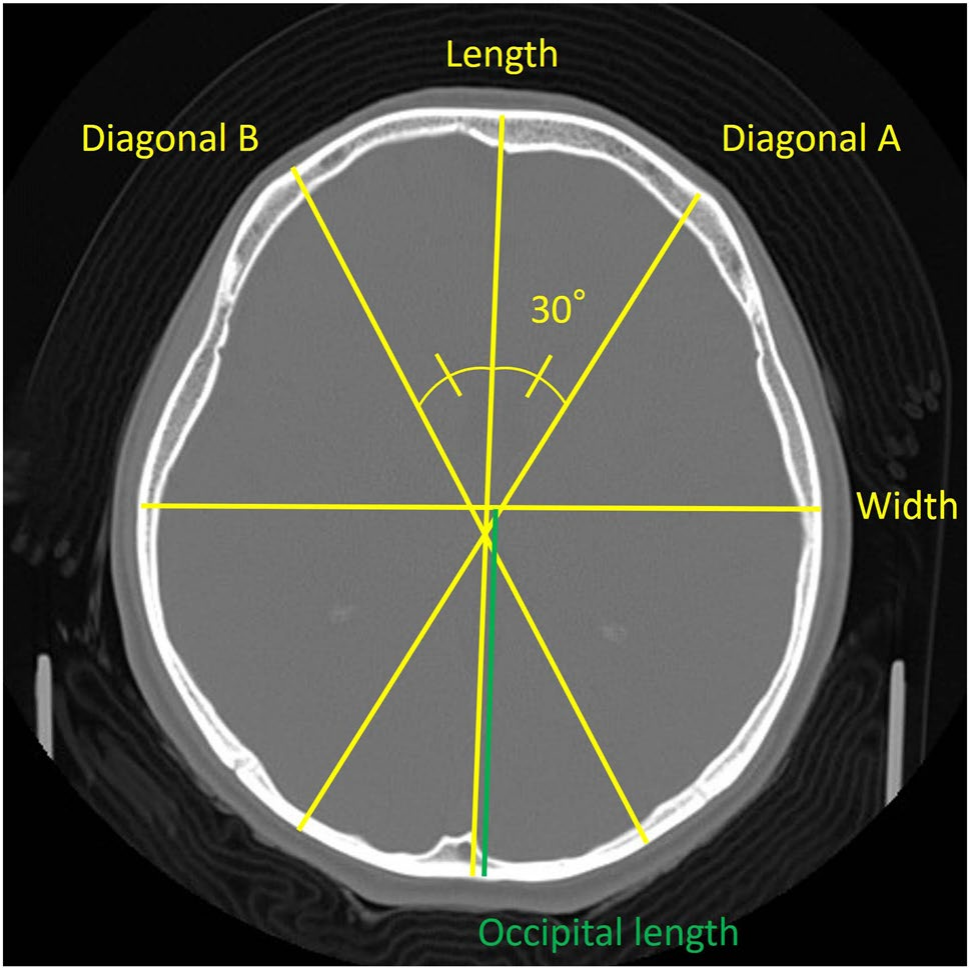
Cranial measurements and evaluation of skull deformities. $$\begin{aligned} {\text{cephalic}}\,{\text{index}}\,\left( {{\text{CI}}} \right) & = \frac{{{\text{width}}}}{{{\text{length}}}} \times 100 \\ {\text{cranial}}\,{\text{vault}}\,{\text{asymmetry}}\,\left( {{\text{CVA}}} \right) \, & = \, \left| {{\text{diagonal}}\,{\text{A}}{-}{\text{diagonal}}\,{\text{B}}} \right| \\ {\text{Flat}}\,{\text{head}}\,{\text{index}}\,\left( {{\text{FHI}}} \right) & = \frac{{{\text{occipital}}\,{\text{length}}\,\left( {{\text{green}}\,{\text{line}}} \right)}}{{{\text{length}}}} \times 100 \\ \end{aligned}$$.

Bone window CT scanning was obtained with an axial slice thickness of 5 mm. The orbit meatal baseline was used as the imaging reference line. The following anthropometric values were measured on the slice including a maximum occipitofrontal diameter length: cranial length, cranial width, the 30 degree angle oblique diagonals (Diag A; right down oblique line, Diag B; left down oblique line), and maximum cranial vault thickness (CVT) of the frontal and occipital bones. Cephalic index (CI) (cranial width/cranial length × 100), the oblique diagonal difference (cranial vault asymmetry (CVA): |Diag A–B|), and the difference between frontal and occipital CVT (occipital CVT—frontal CVT) were calculated. Occipital flattening was evaluated by the composition ratio of the occipital part in cranial length (the distance from intersection of measurement lines for cranial length and width to occipital bone/cranial length × 100), and which was analyzed as Flat head index (FHI) in this study (Fig. 1). These measurements in cases with moyamoya disease, cervical ICA stenosis, and mental diseases were analyzed using head injury cases as control subjects. All data were procured from the medical records of the Dokkyo Medical University Saitama Medical Center. The study was approved by the Medical Ethics Committee of Dokkyo Medical University Saitama Medical Center (based on the principles detailed in the Declaration of Helsinki), and written informed consent was waived because of the study's retrospective design by the Medical Ethics Committee of Dokkyo Medical University Saitama Medical Center. Potential and enrolled research participants were provided information about this research through our website. All procedures were carried out in accordance with the relevant guidelines and regulations.

### Statistical analyses

The chi-squared test or Fisher’s exact test were used to assess group differences in the analysis of qualitative features. The Kruskal–Wallis test was used for a pairwise comparison between the groups. Factors possibly related to head sharp deformities were assessed by univariate and multivariate analyses. Pearson’s correlation was used for an analysis of the correlation between the two variables. A P-value < 0.05 was considered statistically significant.

## Results

### Skull shape by age group in the Japanese population

**Figure 2:**
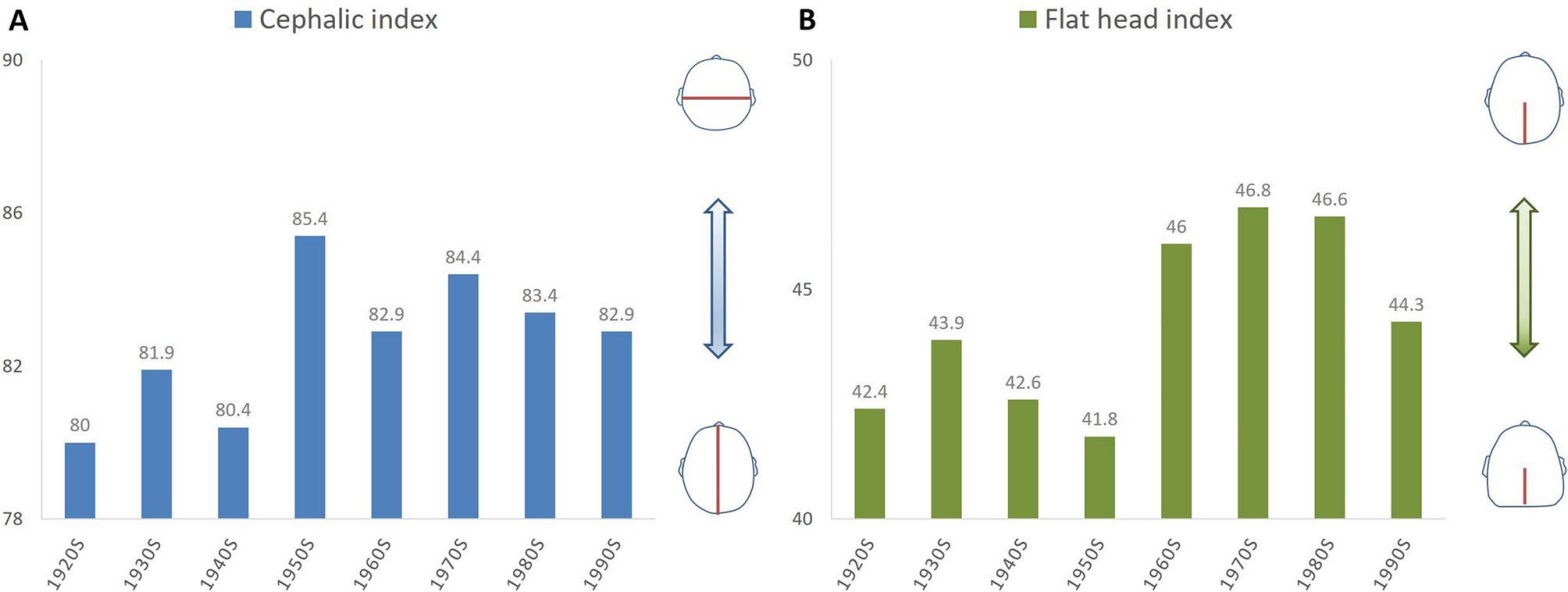
Average values of cephalic index (**A**) and flat head index (**B**) by birth year. People born from the 1950s onwards showed a shorter cranial length, and those born until the 1950s tend to display a stronger occipital flattening.

In head injury cases, the age at the time of receiving the CT scan ranged from 20 to 96 years (median, 56 years), and the sex ratio (male/female) was 2.4. FHI were significantly different across birth years in both univariate and multivariate analyses (*P* < 0.001 and = 0.003, respectively). Significant difference across birth years in CI was obtained in univariate analysis but not in multivariate analysis (*P* = 0.005 and = 0.06, respectively). People born from the 1950s onwards showed shorter cranial lengths than those born until the 1940s. On the other hand, people born until the 1950s tended to have a flattened occipital head comparing to those born from the 1960s onwards (Fig. 2). Significant differences between birth years were seen in CVA and cranial width in univariate analysis (*P* = 0.05 and < 0.001, respectively), but not in multivariate analysis. Anatomic distortion was frequently observed in people born from the 1950s onwards, and a shorter cranial width was seen in people born in the 1920s.

No significant differences in bone thickness were seen between birth years. Moreover, an increase in average thickness was not seen in frontal or occipital bones with aging. On the other hand, there was a tendency for stronger occipital flattening, shorter cranial width, and oval shape with less distortion with aging. A comparison of gender differences indicated a shorter cranial length and width in females, while no differences were shown in CI, FHI, and CVA. Although the occipital bone of males was significantly thicker than that of females (average thickness of male and female was 7.88 and 7.22 mm, respectively, *P* = 0.01), the male frontal bone was thinner than the female one (6.33 and 6.76 mm, respectively). There was a smaller difference between the thickness of frontal and occipital bone in females comparing to that in males.

### Cranial shape variation in cases with moyamoya disease, ICA stenosis, and mental diseases

**Figure 3:**
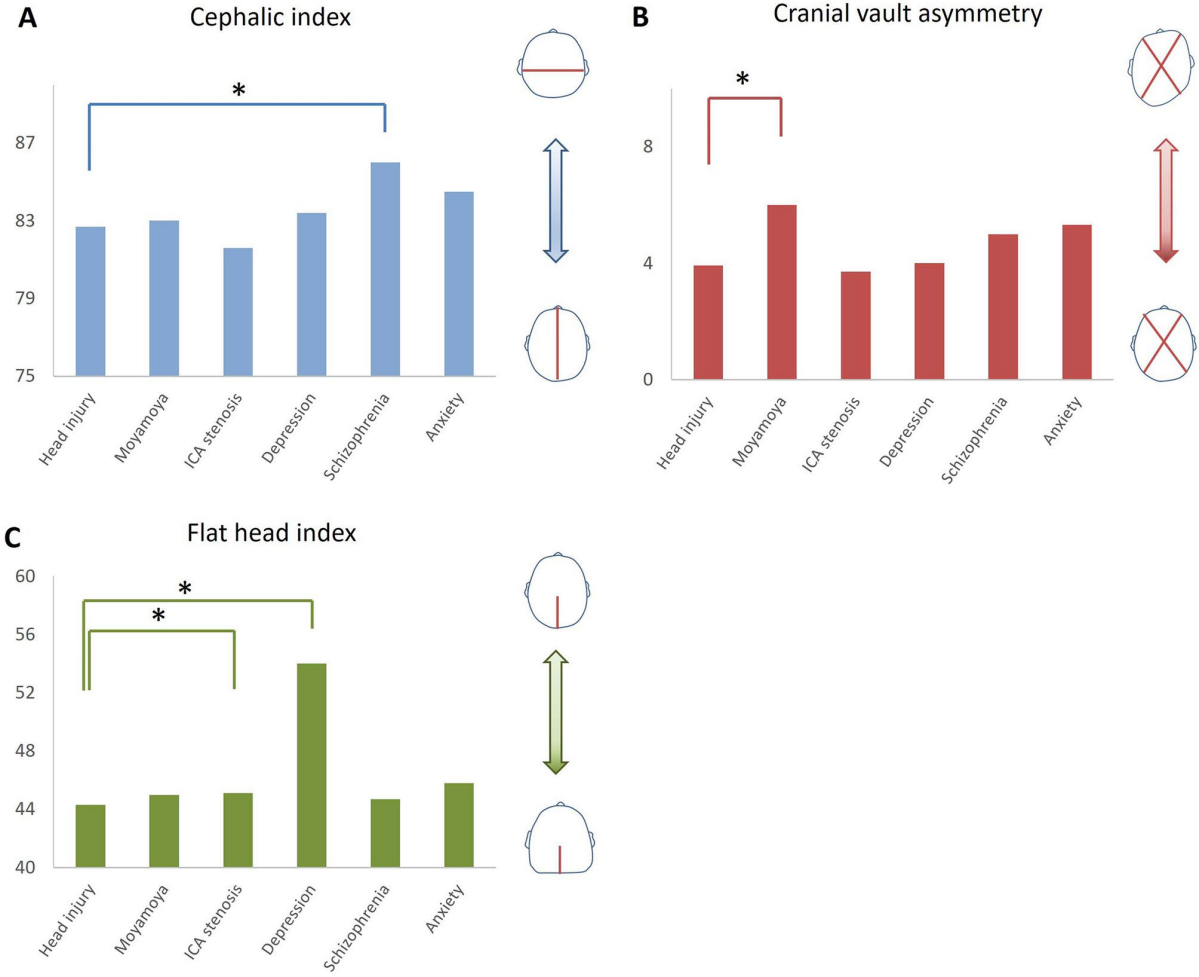
Skull shape assessments in each disease. The bar graph represents values of cephalic index (**A**), cranial vault asymmetry (**B**) and flat head index (**C**) in each disease. Significant differences compared with control subjects (head injury) are indicated with an asterisk (*).

Comparisons of cases with moyamoya disease, cervical ICA stenosis, or mental diseases and cases with head injury as control subjects were reviewed on Table 1 and Fig. 3. Both frontal and occipital bones in moyamoya disease, cervical ICA stenosis, and all mental disease subtypes were significantly thicker than control subjects. The average skull thickness in moyamoya disease was the highest among all subjects. The occipital bone was significantly thicker than the frontal bone in cases with cervical ICA stenosis (*P* < 0.001) and depression (*P* = 0.03). The ratio of occipital CVT to frontal CVT in these two diseases was significantly higher than that of control subjects. On the other hand, the skull in moyamoya disease undergoes equal thickening across the cranial vault, which results in a lower ratio of occipital CVT to frontal CVT compared to control subjects. Differences between left and right bone thickness were investigated in cases with cervical ICA stenosis, to assess whether stenosis side effects bone thickness. Cervical ICA stenosis was observed in 23 cases in the right side, 16 cases in the left side, and 11 cases bilaterally. No significant differences in bone thickness were obtained between severe stenosis side and a no severe stenosis side. Seventeen cases with moyamoya disease revealed laterality in arterial stenosis. Additionally, these cases did not show an association between the degree of bone thickness and severe stenosis.

**Table 1:** The average value in a set of physical and cranial measurments according to primary diseases.

	Head injuries (control subject)	Moyamoya disease	Carotid artery stenosis	Mental disease		
				Depression	Schizophrenia	Anxiety
Case number	160	50	50	50	34	21
Age	54.9	48.2	73.4	60.7	54.5	47.2
Sex (male; female)	113; 47	17; 33	47; 3	19; 31	18; 16	8; 13
Height	163.2	159	161.1	159.4	162.2	161.9
Body weight	59.7	62	59.6	56.4	61.5	58.7
**Cranial measurments**						
Length	178.2	178	180.1	176.4	173.7*,**	178.2
Width	147.2	148	146.9	147	149.2**	150.5**
O. length	79.1	80	81.3**	95.6*,**	77.8	81.7
Diagonal A	169.3	159*,**	165.7	166.3	166	167.5
Diagonal B	169.7	160*,**	166	165.2	167.1	171.3
F. CVT	6.46	10*,**	8.27*,**	8.74*,**	8.81*,**	8.98*,**
O. CVT	7.69	11*,**	10.4*,**	10.1*,**	9.93*,**	10*,**
**Assessment of skull shape**						
CI	82.7	83	81.6	83.4	86*,**	84.5
CVA	3.93	6*,**	3.71	4	5	5.33
FHI	44.3	45	45.1**	54*,**	44.7	45.8
O–F CVT	1.23	0.66*	2.12*,**	1.42**	1.11	1.08

*CI* cephalic index, *CVA* cranial vault asymmetry, *CVT* cranial vault thickness, *F* frontal, *FHI* flat head index, *O* occipital, *O–F* occipital–frontal, Notes showing significant difference (P < 0.05%) comparing to control subjects in univariate (*) and multivariate (**) analysis.

**Figure 4:**
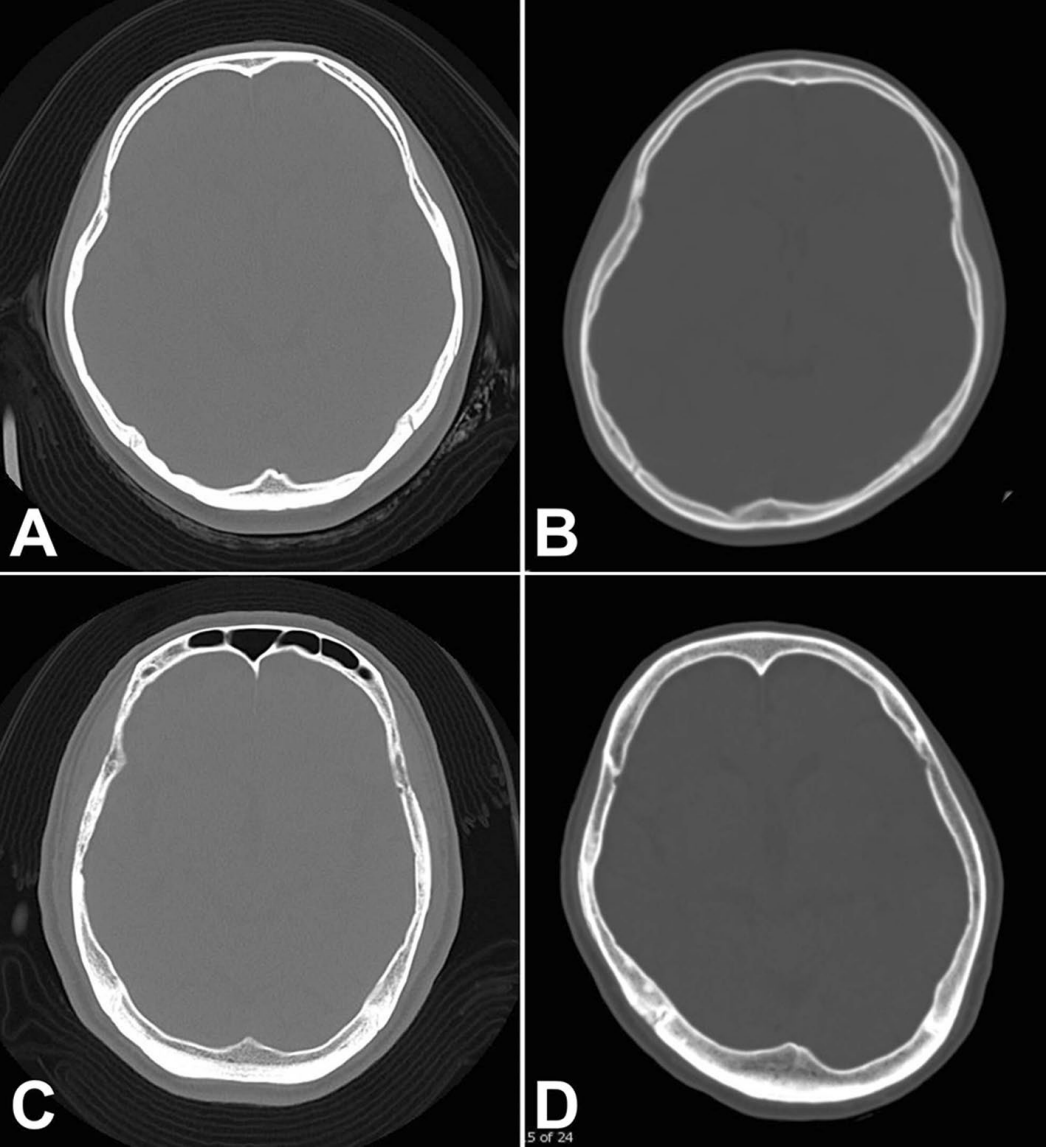
Representative computed tomography scan findings. An axial computed tomography scan of the head with a bone window from head injury (**A**), moyamoya disease (**B**), cervical internal carotid artery stenosis (**C**), and depression (**D**).

On analysis of cranial shape deformity, significantly longer occipital length and subsequently higher FHI values were seen in cases with cervical ICA stenosis (*P* < 0.001) and depression (*P* = 0.03). A shorter cranial length, longer width, and subsequently higher CI were seen in cases with schizophrenia (*P* < 0.001). Cases with moyamoya disease showed significantly shorter oblique diagonals (*P* < 0.001) and higher CVA (*P* = 0.002). In fact, plagiocephaly showing a CVA of 5 mm or more was seen in 21 of 35 (60%) cases with moyamoya disease, and in 54 of 160 (34%) cases with head injuries (odds ratio [OR], 0.34; 95% confidence interval, 0.16–0.72; *P* = 0.006). The degree of cranial shape deformity (CVA and CI) did not correlate to that of bone thickness in moyamoya disease. Representative CT scans of each primary disease are showed in Fig. 4.

## Discussion

The average thickness of the frontal and occipital bones is 8 mm, while the temporal bone is the thinnest in the cranial vault with an average thickness of 4 mm^14^. The bone is constantly remodeling through tightly coupled bone resorption and formation. Healthily aging adults normally experience a decrease in cortical bone with an increase in trabecular porosity, and the average skull thickness of the total cranial vault remains unchanged with age after the head circumference reaches a plateau^15^. Our subjects from age 20 to 96 years of age showed no age-related changes in average skull thickness. In Japanese people, there were significant differences in FHI across birth years. People born from the 1950s onwards showed a brachiocephalic skull and people born in the 1990s and 1920–1950s showed an occipital flattening tendency. Japanese babies are traditionally put to sleep lying on their backs, on a pillow and a Japanese-style mattress. Mattresses made from a hard material had been commonly used until the 1950s, while baby buggies and soft mattresses became widespread from the 1960s. In the 1990s, the campaign advising parents to put their baby sleeping on their back started in order to avoid sudden infant death syndrome. These historical backgrounds may be associated with an occipital flattening tendency in specific Japanese generations.

Calvarial thickening can occur from several diseases such as acromegaly, Paget disease, fibrous dysplasia, osteopetrosis, and chronic ventricular shunting or phenytoin usage^16–18^. Cadaver studies identified that the thickening of the skull, particularly in the frontal area was observed in 29% of cases, and 26% of them, had neuropsychiatric disorders including Alzheimer’s, dementia, depression, and Parkinson’s disease^19^. On the other hand, few studies have focused on skull shape changes in patients with cerebrovascular or mental diseases. Our analysis identified that diseases causing a chronic reduction in cerebral blood flow were associated with calvarial thickening. Calvarial thickening was seen both in moyamoya and cervical ICA stenosis, which implies that a chronic reduction in cerebral blood causes calvarial thickening in both children and adults, along with an increase in blood supply from the external carotid artery. It has been widely recognized that rich blood supply is associated with bone growth and maturity^20,21^. The skull mainly receives arterial blood supply via the external carotid artery^22^. Interestingly, moyamoya disease revealed a thicker calvarium than other diseases, and displayed more prominent frontal bone thickening than other regions. In addition, plagiocephaly was more frequently observed in moyamoya disease. These results suggest that increasing blood supply to the skull during the growth period leads to early skeletal maturity, consequent partial synostosis, and disproportional calvarial thickening by partial microvascular development.

Significant calvarial thickening was also observed in all mental disease cases analyzed, and schizophrenia additionally revealed a brachycephalic tendency. Depression cases showed a high occipital length, distinct from flat head syndrome. Although plagiocephaly in babies has been reported to be associated with a mental or psychomotor developmental delay, our results did not prove the hypothesis that persistent plagiocephaly in adults is associated with mental disorders. On the other hand, a strong association was identified between mental diseases and calvarial thickening. Large numbers of neuroimaging studies have revealed a reduction in regional gray matter volume with a significant reduction in cerebral blood volume in general anxiety disorders, depression, and schizophrenia^23–29^. The long disease course correlates with a more severe reduction in cerebral blood volume. The cerebral blood flow is reduced when supplying a certain amount of circulating blood to the head and neck, which increases blood flow volume of the external carotid artery, and thereby developing calvarial thickening in these mental diseases. In summary, significant calvarial thickening was observed in adult cases with moyamoya disease, cervical ICA stenosis, and mental diseases. Moyamoya disease and schizophrenia revealed a tendency for plagiocephaly and brachycephaly, respectively. CT is a simple and inexpensive examination, which is commonly used as the first step in screening for neurological diseases. We suggest that these uncommon skull shapes are useful CT findings in screening subjects for early evidence of mental diseases and intracranial ischemic diseases with arterial stenosis.

## Data Availability

The datasets generated during and/or analysed during the current study are available from the corresponding author on reasonable request.
